# SCD1 Sustains Homeostasis of Bulge Niche via Maintaining Hemidesmosomes in Basal Keratinocytes

**DOI:** 10.1002/advs.202201949

**Published:** 2022-12-11

**Authors:** Yueqing Xue, Liangyu Lin, Qing Li, Keli Liu, Mingyuan Hu, Jiayin Ye, Jianchang Cao, Jingjie Zhai, Fanjun Zheng, Yu Wang, Tao Zhang, Liming Du, Cheng Gao, Guan Wang, Xuefeng Wang, Jun Qin, Xinhua Liao, Xiangyin Kong, Lydia Sorokin, Yufang Shi, Ying Wang

**Affiliations:** ^1^ CAS Key Laboratory of Tissue Microenvironment and Tumor Shanghai Institute of Nutrition and Health University of Chinese Academy of Sciences Chinese Academy of Sciences Shanghai 200031 China; ^2^ The Third Affiliated Hospital of Soochow University State Key Laboratory of Radiation Medicine and Protection, Institutes for Translational Medicine Soochow University Medical College Suzhou Jiangsu 215123 China; ^3^ Institute of Physiological Chemistry and Pathobiochemistry Cells in Motion Interfaculty Centre (CIMIC) University of Münster D‐48149 Münster Germany; ^4^ School of Life Sciences Shanghai University Shanghai 200444 China

**Keywords:** bulge, hair follicle stem cells, hemidesmosomes, keratinocytes, stearoyl‐CoA desaturase 1

## Abstract

Niche for stem cells profoundly influences their maintenance and fate during tissue homeostasis and pathological disorders; however, the underlying mechanisms and tissue‐specific features remain poorly understood. Here, it is reported that fatty acid desaturation catabolized by stearoyl‐coenzyme A desaturase 1 (SCD1) regulates hair follicle stem cells (HFSCs) and hair growth by maintaining the bulge, niche for HFSCs. *Scd1* deletion in mice results in abnormal hair growth, an effect exerted directly on keratin K14^+^ keratinocytes rather than on HFSCs. Mechanistically, *Scd1* deficiency impairs the level of integrin *α*6*β4* complex and thus the assembly of hemidesmosomes (HDs). The disruption of HDs allows the aberrant activation of focal adhesion kinase and PI3K in K14^+^ keratinocytes and subsequently their differentiation and proliferation. The overgrowth of basal keratinocytes results in downward extension of the outer root sheath and interruption of bulge formation. Then, inhibition of PI3K signaling in *Scd1^−/−^* mice normalizes the bulge, HFSCs, and hair growth. Additionally, supplementation of oleic acid to *Scd1^−/−^* mice reestablishes HDs and the homeostasis of bulge niche, and restores hair growth. Thus, SCD1 is critical in regulating hair growth through stabilizing HDs in basal keratinocytes and thus sustaining bulge for HFSC residence and periodic activity.

## Graphical abstract

Fatty acid metabolism can regulate the hair growth by maintaining the bulge, niche for hair follicle stem cells. Mechanistically, *Scd1* deficiency impairs the assembly of hemidesmosomes, which causes the aberrant differentiation and proliferation of keratin K14^+^ keratinocytes. The overgrowth of basal keratinocytes results in downward extension of the outer root sheath and interruption of bulge formation.

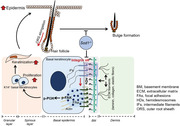

## 1 Introduction

The stem cell niche serves as an anatomical structure to provide appropriate spatiotemporal signals for maintaining the balance between the quiescent and active states of stem cells.^[^ ^1^ ^]^ Multiple niche components are essential for safeguarding stem cells to respond to appropriate stimuli that regulate stem cell reserve and activation. Recent investigations have identified niches for positioning various stem cells,^[^ ^2^ ^]^ however, the key mechanisms that govern the formation of stem cell niches remain largely unknown.

The hair follicle (HF) bulge is a specialized niche that maintains hair follicle stem cells (HFSCs) in position to properly function during the hair regeneration.^[^ ^3^, ^4^ ^]^ Absence of the bulge leads to hair loss.^[^ ^5^ ^]^ During hair growth, HFs undergo periodic waves of anagen (growth), catagen (destruction), and telogen (resting). At early anagen, HFSCs transiently exit their quiescent status to proliferate and migrate downward (out of the bulge), giving rise to the outer root sheath (ORS) keratinocytes with stem cell markers such as SOX9 and Lgr5 to envelop the elongated HFs.^[^ ^6^, ^7^ ^]^ At catagen stage, the cells in the upper ORS upregulate CD34 expression and form a new bulge adjacent to the original one to reserve HFSCs for the next hair cycle.^[^ ^8^, ^9^ ^]^

Extensive studies have demonstrated the intrinsic factors regulating the activity of HFSCs during hair growth. The activity of HFSCs can be controlled by multiple cell types, like fibroblasts, adipocytes, and certain types of immune cells in the bulge and the skin microenvironment, and by their associated molecules, such as BMPs, Wnt, SHH, and PDGF.^[^ ^10^, ^11^, ^12^, ^13^, ^14^, ^15^ ^]^ Disruption of bulge signals could lead to a failure in maintaining intact HFs with quiescent HFSCs.^[^ ^1^, ^9^, ^16^ ^]^ Thus, the bulge niche is vital for appropriately controlling HFSC position and activity. However, the key mechanisms governing the bulge formation and maintenance remain unclear.

By dissecting the metabolic traits of HFSCs during hair growth, studies have revealed that a secondary bulge formation was related to the suppression of metabolic switch in HFSCs from glycolysis to oxidative phosphorylation (OXPHOS) and glutamine metabolism, allowing the reversibility of ORS cells into HFSCs in the quiescent state and thus promoting HF regeneration.^[^ ^8^, ^17^, ^18^ ^]^ Our previous study in adipocytes found that deficiency of stearoyl‐coenzyme A desaturase 1 (*Scd1*), a rate‐limiting enzyme in maintaining the balance between saturated fatty acids and monounsaturated fatty acids, can expedite OXPHOS and glutamine metabolism.^[^ ^19^ ^]^ We questioned whether targeting fatty acid metabolism could regulate the bulge niche formation, HFSCs activities, and hair growth.

Consistent with previous observations, loss of *Scd1* resulted in a series of cutaneous abnormalities, including alopecia.^[^ ^20^, ^21^, ^22^ ^]^ Here, we demonstrate that *Scd1* deficiency did not directly influence the number and activity of HFSCs, but rather impaired the formation of the first bulge during the postnatal period, leading to the depletion of HFSC reservation during hair growth. We show that alteration of *Scd1* expression in keratinocytes alone leads to the attenuation of integrin *α*6*β*4 mediated hemidesmosome (HD) formation in K14^+^ basal keratinocytes. The reduction in HD was associated with enhanced proliferation and differentiation of basal keratinocytes through activating the PI3K signaling pathway in a focal adhesion kinase (FAK)‐dependent manner. These changes in basal keratinocytes eventually blocked the formation of the bulge niche and reservation of quiescent HFSCs. Our investigations not only provide a mechanistic insight into how fatty acid metabolism in the skin tissue microenvironment regulates the homeostasis of the bulge niche for HFSCs, but also open avenues toward new strategies to combat cutaneous disorders.

## 2 Results

### 2.1 *Scd1* Deficiency Impairs the Homeostasis of Bulge Niche for HFSCs

**Figure 1:**
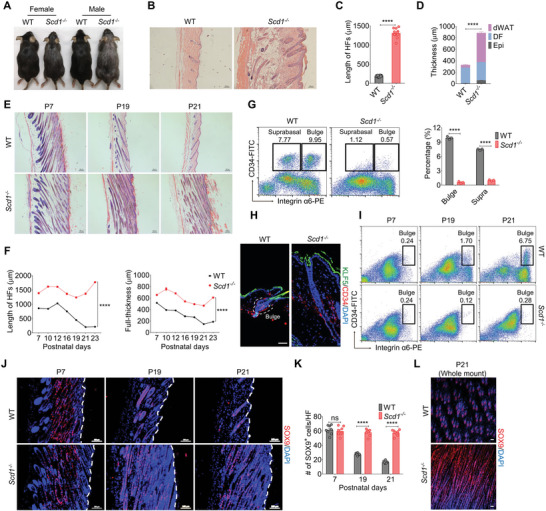
*Scd1* deficiency impairs the homeostasis of the bulge niche for HFSCs. A) Hair coat of female and male wild‐type (WT) and *Scd1^−/−^* mice. B) Representative images of H&E staining of skin sections of adult WT and *Scd1^−/−^* mice. Scale bars: 100 µm. C,D) Quantification of the length of HFs (C) (*n* = 12; two‐tailed unpaired *t*‐test) and thickness of the epidermis (Epi), dermal fibroblast (DF), and subcutaneous adipocytes (dWAT) (D) (*n* = 6; two‐tailed unpaired *t*‐test) in WT and *Scd1^−/−^* mice. E) Representative images of H&E staining of skin sections of WT and *Scd1^−/−^* mice in the three phases of the first hair cycle: anagen (postnatal 7 days, P7), catagen (postnatal 19 days, P19), and telogen (postnatal 21 days, P21). Scale bars: 100 µm. F) Quantification of the length of HFs (left) (*n* = 6; two‐way ANOVA with Sidak's two‐sided multiple comparisons) and full‐thickness of skin (right) (*n* = 6; two‐way ANOVA with Sidak's two‐sided multiple comparisons) of skin sections of WT and *Scd1^−/−^* mice at different time points of the first hair cycle. G) The percentage of HFSCs in WT and *Scd1^−/−^* mice as analyzed by flow cytometry, including integrin *α*6^high^CD34^+^ HFSCs and integrin *α*6^low^CD34^+^ suprabasal population (*n* = 3; two‐tailed unpaired *t*‐test). H) Immunostaining of CD34 and KLF5 in keratinocytes of WT and *Scd1^−/−^* mice. Red: CD34; green: KLF5; blue: DAPI. Scale bars: 50 µm. I) The percentage of integrin *α*6^high^CD34^+^ bulge HFSCs in epidermal cells harvested from WT and *Scd1^−/−^* mice during the first hair cycle. J) Immunostaining of SOX9 in the skin of WT and *Scd1^−/−^* mice during the three phases of the first hair cycle: anagen (P7), catagen (P19), and telogen (P21). Red: SOX9; blue: DAPI. The white dotted line indicates the basal layer of epidermis. Scale bars: 100 µm. K) Quantification of SOX9^+^ cells per HF in panel (J) (*n* = 8; two‐way ANOVA with Sidak's two‐sided multiple comparisons). L) Representative skin whole‐mount images of bulge formation in WT and *Scd1^−/−^* mice at telogen stage (P21) as determined by staining with a SOX9‐specific antibody. Scale bars: 20 µm. Data are shown as mean ± SEM. ****p < 0.0001; ns, not significant.

We first examined the expression of *Scd* isoforms in the mouse skin. Among them, SCD1 is the most predominant isoform and its transcript levels in the skin tissue fluctuated along with the hair cycle stages during physiological or depilation‐induced regeneration (Figure S1A–C, Supporting Information). Consistent with previous studies, loss‐of‐function mutation or genetic deletion of *Scd1* (*Scd1^−/−^*) in mice lead to the alopecia phenotype (**Figure** **1**A).^[^ ^21^, ^22^, ^23^ ^]^ However, the mechanisms that *Scd1* deficiency induces alopecia remain largely unexplored. Histological analysis showed that *Scd1* deficiency did not affect the density of HFs; rather their length was significantly elongated (Figure 1B,C). We also noticed that the epidermis and subcutaneous adipocyte layer in *Scd1^−/−^* mice were dramatically thickened (Figure 1B,D). It is known that the HFs undergo morphological changes during the hair cycle.^[^ ^24^ ^]^ To compare the morphology of HFs in wild‐type (WT) and *Scd1^−/−^* mice, we analyzed the HFs at different stages of the first hair cycle in the skin samples from *Scd1^−/−^* mice and their littermate controls. We found that the HFs were elongated in *Scd1^−/−^* mice, and the skin was thickened even at telogen stage (P21) (Figure 1E,F). To further compare the morphology of HFs between WT and *Scd1^−/−^* mice, the skin samples were placed subcutaneous adipocytes side up and examined under the stereomicroscope (Figure S1D, Supporting Information). Combined with the hematoxylin and eosin (H&E) staining, the HFs in the skin of WT mice presented periodical changes, according to the hair cycle stages.^[^ ^24^ ^]^ However, the HFs and hair shafts of *Scd1^−/−^* mice were extended into subcutaneous adipocyte layer, suggesting that the deletion of *Scd1* affected the hair growth cycle.

To determine the cellular mechanisms of the altered HFs and hair growth in adult *Scd1^−/−^* mice, we examined HFSCs by flow cytometry. It is believed that the purified label‐retaining cells in the HFs rarely divide within their niche and are featured by the expressions of integrin *α*6 (also known as CD49f) and CD34.^[^ ^25^ ^]^ Both integrin *α*6^high^CD34^+^ and integrin *α*6^low^CD34^+^ cells represent the stem cell population.^[^ ^26^ ^]^ We found that both of them were significantly reduced in *Scd1^−/−^* mice as compared to that in WT mice (Figure 1G). Meanwhile, immunofluorescence staining revealed that fewer CD34^+^ cells in the bulge were observed in *Scd1^−/−^* mice than in WT mice (Figure 1H). Moreover, the reduction in integrin *α*6^high^CD34^+^ bulge HFSCs was seen even at the first telogen stage in *Scd1^−/−^* mice (Figure 1I). Therefore, we questioned whether HFSCs were sustainably mobilized and unable to return to the quiescence stage due to the low expression in integrin *α*6. According to integrin *α*6^high^CD34^+^ bulge HFSCs can also come from cells of the upper ORS during catagen to telogen transition,^[^ ^6^ ^]^ we immunostained skin sections from different hair cycle stages with an antibody specific to SOX9, a marker of ORS cells, to assess whether the reduction of HFSCs in *Scd1^−/−^* mice was due to the changes in ORS.^[^ ^6^, ^12^ ^]^ We found that the SOX9^+^ ORS remained elongated and was unable to form the bulge niche to reserve the quiescent HFSCs at the telogen stage (P21) in *Scd1^−/−^* mice in skin sections staining or whole‐mount staining (Figure 1J–L). Loss of the first bulge formation affected subsequent hair cycles, raising an explanation for the deficiency of integrin *α*6^low^CD34^+^ suprabasal bulge cells (Figure 1G). Therefore, the absence of *Scd1* impacts the formation of bulge for HFSCs and thus prohibits the normal hair cycle.

### 2.2 Hair Loss in *Scd1^−/−^* Mice Is not Directly Related to Intrinsic Changes in HFSCs

**Figure 2:**
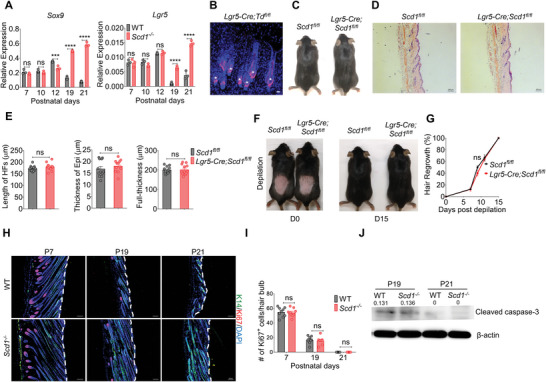
Hair loss in *Scd1^−/−^* mice is not directly related to intrinsic changes in HFSCs. A) The expression of *Sox9* (left) (n = 3; two‐tailed unpaired *t*‐test) and *Lgr5* (right) (*n* = 3; two‐tailed unpaired *t*‐test) in the skin of WT and *Scd1^−/−^* mice at different time points of the first hair cycle as detected by RT‐PCR. B) Representative skin whole‐mount images of Lgr5^+^ cells in HFs of mice with HFSCs‐specific tracking system, *Lgr5‐Cre;Td^fl/fl^*. Red: Td; blue: DAPI. Scale bars: 20 µm. C) The hair coat of mice with *Scd1*‐specific knockout in HFSCs (*Lgr5‐Cre;Scd1^fl/fl^* mice) compared with the littermate controls (*Scd1^fl/fl^* mice). D) Representative images of H&E staining of the skin of *Lgr5‐Cre;Scd1^fl/fl^* mice and littermate controls. Scale bars: 100 µm. E) Quantification of the length of HFs (left) (*n* = 12; two‐tailed unpaired *t*‐test), thickness of epidermis (middle) (*n* = 12; two‐tailed unpaired *t*‐test) and the full‐thickness of skin (right) (*n* = 12; two‐tailed unpaired *t*‐test) in panel (D). F) The hair coat of *Lgr5‐Cre;Scd1^fl/fl^* mice and *Scd1^fl/fl^* mice on day 0 (left) and 15 (right) post hair plunking. G) Kinetics of hair regrowth of *Lgr5‐Cre;Scd1^fl/fl^* mice and littermate controls after depilation (*n* = 4; two‐tailed unpaired *t*‐test). H) Immunostaining of Ki67 in the skin of WT and *Scd1^−/−^* mice in the three phases of the first hair cycle: anagen (P7), catagen (P19), and telogen (P21). Red: Ki67; green: K14; blue: DAPI. The white dotted line indicates the epidermis. Scale bars: 100 µm. I) Quantification of Ki67^+^ cells in the hair bulb during the three phases of the first hair cycle: anagen (P7), catagen (P19), and telogen (P21) (*n* = 9; two‐way ANOVA with Sidak's two‐sided multiple comparisons). J) Western blotting analysis of cleaved caspase‐3 in the skin of WT and *Scd1^−/−^* mice at catagen (P19), telogen (P21) phases of the first hair cycle. Data are shown as mean ± SEM. ***p < 0.001; ****p < 0.0001; ns, not significant.

The proper appearance and timely behavior of HFSCs are directly associated with hair growth, and their abnormality causes hair loss.^[^ ^5^ ^]^ Next, we monitored the expression of *Lgr5* and *Sox9*, markers of HFSCs, in the skin at different time points during the first hair cycle and found that they maintained at high levels even at catagen (postnatal day 19, P19) and telogen (postnatal day 21, P21) stages in *Scd1^−/−^* mice (**Figure** **2**A), suggesting that *Scd1* deficiency may even promote the mobilization of HFSCs. To directly test the effect of SCD1 on HFSCs, we generated *Lgr5‐Cre;Scd1^fl/fl^* mice to delete *Scd1* in HFSCs (Figure 2B; Figure S2A,B, Supporting Information). These *Lgr5‐Cre;Scd1^fl/fl^* mice had normal hair coat (Figure 2C), demonstrating that the effect of SCD1 on hair growth is not exerted through HFSCs. In addition, such specific deletion of *Scd1* in HFSCs did not affect the morphology of HFs, epidermis and dermis (Figure 2D,E). Furthermore, when hair regrowth after depilation was tested, there was no difference between *Lgr5‐Cre;Scd1^fl/fl^* mice and littermate controls (Figure 2F,G).

To reconcile the different hair coat phenotypes of *Scd1^−/−^* mice and *Lgr5‐Cre;Scd1^fl/fl^* mice, we evaluated the proliferation and apoptosis of the cells in hair bulb to understand the effect of SCD1 on growth and regression of HFSCs, respectively.^[^ ^24^ ^]^ Surprisingly, in *Scd1^−/−^* mice, although there are elongated HFs, the number of Ki67^+^ proliferating cells and apoptotic cells in hair bulbs were comparable to those in WT mice (Figure 2H–J), suggesting that the HF defects observed may be indirect effects. These results corroborated with the observation made from *Lgr5‐Cre;Scd1^fl/fl^* mice, demonstrating that the abnormal hair growth in *Scd1^−/−^* mice is not related to the SCD1 status in HFSCs.

### 2.3 SCD1 in K14^+^ Basal Keratinocytes Is Required for Sustaining the Bulge for HFSCs

**Figure 3:**
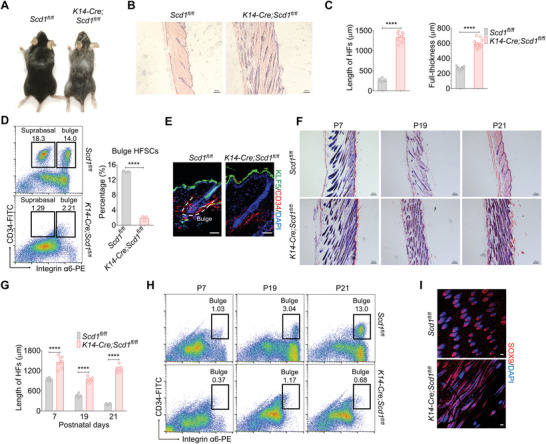
SCD1 in K14^+^ basal keratinocytes is required for sustaining the bulge for HFSCs. A) Hair coat of mice with K14‐specific deletion of SCD1 (*K14‐Cre;Scd1^fl/fl^*) and littermate controls (*Scd1^fl/fl^* mice). B) Representative images of H&E staining of skin sections of *K14‐Cre;Scd1^fl/fl^* mice and littermate controls. Scale bars: 100 µm. C) Quantification of the length of HFs (*n* = 12; two‐tailed unpaired *t*‐test) and full‐thickness of the skin (*n* = 16; two‐tailed unpaired *t*‐test) of *K14‐Cre;Scd1^fl/fl^* mice and littermate controls. D) The percentage of the HFSCs analyzed by flow cytometry in *K14‐Cre;Scd1^fl/fl^* mice and littermate controls (*n* = 3; two‐tailed unpaired *t*‐test). E) Immunostaining of CD34 and KLF5 in keratinocytes of WT and *K14‐Cre;Scd1^fl/fl^* mice. Red: CD34; green: KLF5; blue: DAPI. Scale bars: 50 µm. F) Representative images of H&E staining of skin sections of *K14‐Cre;Scd1^fl/fl^* mice and littermate controls in the three phases of the first hair cycle: anagen (P7), catagen (P19), and telogen (P21). Scale bars: 100 µm. G) Quantification of the length of HFs of *K14‐Cre;Scd1^fl/fl^* mice and littermate controls at different time points of the first hair cycle (*n* = 8; two‐tailed unpaired *t*‐test). H) The percentage of HFSCs in *K14‐Cre;Scd1^fl/fl^* mice, and *Scd1^fl/fl^* mice during the first hair cycle. I) Representative skin whole‐mount images of bulge formation in *K14‐Cre;Scd1^fl/fl^* mice and *Scd1^fl/fl^* mice at telogen stage (P21) as determined by staining with a SOX9 specific antibody. Scale bars: 20 µm. Data are shown as mean ± SEM. ****p < 0.0001.

To investigate the effects of SCD1 on other cellular components in the skin, we examined *Scd1* expression in epidermal cells and HFSCs, and found abundant *Scd1* expression in epidermal cells (Figure S2C, Supporting Information). *Scd1* was subsequently specifically deleted in keratinocytes using Keratin 14 (K14) promoter‐driven recombinase (Figure S2D,E, Supporting Information) and the resultant *K14‐Cre;Scd1^fl/fl^* mice exhibited the same HF defects as observed in *Scd1^−/−^* mice (**Figure** **3**A).^[^ ^27^ ^]^ HFs were elongated and the epidermis was thickened in *K14‐Cre;Scd1^fl/fl^* mice, as seen in *Scd1^−/−^* mice (Figure 3B,C). Furthermore, the number of integrin *α*6^high^CD34^+^ HFSCs was dramatically decreased in *K14‐Cre;Scd1^fl/fl^* mice, as shown in immunostaining (Figure 3D,E). More interestingly, analysis of the first hair cycle of *K14‐Cre;Scd1^fl/fl^* mice showed that ORS did not shorten to form the bulge niche (Figure 3F–I). These results demonstrate that *Scd1* deficiency in K14^+^ keratinocytes drives the cutaneous abnormalities and subsequent hair loss.

Since histological analysis showed that *Scd1* ^−/−^ mice exhibited enhanced immune cell infiltration and thickened subcutaneous adipocyte layer, we wondered whether the hair loss was induced by the changes in the tissue microenvironment. We found that *Scd1^−/−^* mice displayed more severe inflammation in imiquimod (IMQ)‐induced psoriasiform skin pathology than littermate controls (Figure S2F, Supporting Information).^[^ ^28^, ^29^ ^]^ To investigate the potential effects of *Scd1* deficiency on immune cells and adipocytes in the skin microenvironment which have been implicated in HF development,^[^ ^10^, ^11^, ^12^, ^30^, ^31^ ^]^ we also generated *CD4‐Cre;Scd1^fl/fl^* and *Lyz2‐Cre;Scd1^fl/fl^* mice to eliminate *Scd1* expression in cells of the adaptive and innate immune cells, and *Dermo1‐Cre;Scd1^fl/fl^* mice to eliminate *Scd1* expression in dermal stromal cells, which included adipocytes (Figure S2G–M, Supporting Information).^[^ ^19^, ^32^, ^33^, ^34^ ^]^ These mice exhibited normal hair growth and skin structure. Thus, SCD1 in K14^+^ keratinocytes is indispensable in maintaining the cutaneous structure.

### 2.4 Loss of *Scd1* Compromises the Bulge Formation by Provoking PI3K‐Mediated Overproliferation and Hyperkeratinization of Keratinocytes

**Figure 4:**
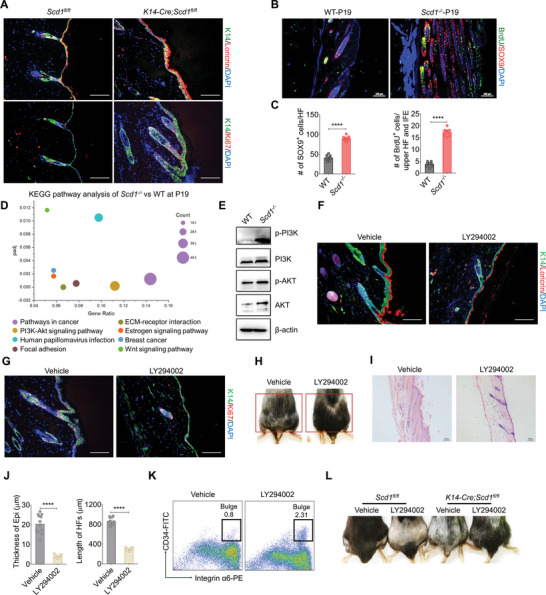
Loss of *Scd1* compromises the bulge formation by provoking PI3K‐mediated overproliferation and hyperkeratinization of keratinocytes. A) Immunostaining of loricrin and Ki67 in skin sections of *K14‐Cre;Scd1^fl/fl^* mice and *Scd1^fl/fl^* mice. Red: loricrin (top), Ki67 (bottom); green: K14; blue: DAPI. Scale bars: 130 µm. B) Immunostaining of SOX9 and BrdU of WT and *Scd1^−/−^* mice. Red: SOX9; green: BrdU; blue: DAPI. Scale bars: 100 µm. C) Quantification of SOX9^+^ (*n* = 9; two‐tailed unpaired *t*‐test) and BrdU^+^ (*n* = 9; two‐tailed unpaired *t*‐test) cells per upper HF of WT and *Scd1^−/−^* mice at P19 in panel (B). D) The top 8 Kyoto Encyclopedia of Genes and Genomes (KEGG) pathways enriched in RNA‐seq analysis of epidermal cells from WT and *Scd1^−/−^* mice at P19. E) Western blotting analysis of the phosphorylation status of PI3K‐Akt signaling in primary keratinocytes isolated from neonatal WT mice and *Scd1^−/−^* mice. F,G) Immunostaining of loricrin (F) or Ki67 (G) in skin sections of *Scd1^−/−^* mice after topical treatments with vehicle control (DMSO in cream base) (*n* = 3) or PI3K inhibitor LY294002 (dissolved in DMSO and then added to cream base) (*n* = 4) every other day for 30 days. Red: loricrin (F), Ki67 (G); green: K14; blue: DAPI. Scale bars: 130 µm. H) Hair growth of *Scd1^−/−^* mice treated with vehicle control or PI3K inhibitor LY294002. I) Representative images of H&E staining of skin sections of *Scd1^−/−^* mice treated with vehicle control or LY294002. Scale bars: 100 µm. J) Quantification of the thickness of the epidermis (*n* = 12; two‐tailed unpaired *t*‐test) and HF length (*n* = 12; two‐tailed unpaired *t*‐test) in panel (I). K) The percentage of integrin *α*6^high^CD34^+^ bulge HFSCs in *Scd1^−/−^* mice treated with vehicle control or LY294002. L) Hair growth of *K14‐Cre;Scd1^fl/fl^* mice and *Scd1^fl/fl^* mice treated with vehicle control or PI3K inhibitor LY294002. Data are shown as mean ± SEM. ****p < 0.0001.

K14^+^ cells exist in both HFs and epidermis of the skin (Figure S2D, Supporting Information). Although the length of HFs was increased in *Scd1^−/−^* mice, the proliferation and apoptosis of K14^+^ cells in the hair bulb of HFs were not altered (Figure 2H–J). Therefore, the abnormities in the interfollicular epidermis (IFE) may be responsible for the dysregulated skin microenvironment and bulge deformation in *Scd1^−/−^* mice. It is well known that K14^+^ basal keratinocytes in IFE differentiate into loricrin^+^ stratum corneum cells.^[^ ^35^, ^36^ ^]^ Consistent with the hyperkeratinized epidermis (Figure S3A,B, Supporting Information), immunofluorescence staining of K14 and loricrin indeed proved that keratinization was enhanced in the skin of *K14‐Cre;Scd1^fl/fl^* mice (**Figure** **4**A, top). Significantly, the proliferation of K14^+^ basal keratinocytes was dramatically increased in these mice (Figure 4A, bottom). Similar results were achieved by loricrin or Ki67 staining in the epidermis of *Scd1^−/−^* mice (Figure S3C–E, Supporting Information). To exclude the possibility that the activation of K14^+^ basal keratinocytes is a secondary effect due to cumulative damage, we analyzed the proliferation and differentiation of K14^+^ keratinocytes during the first hair cycle. Clearly, there are more Ki67^+^ cells in the upper part of the HFs and the epidermis of *Scd1^−/−^* mice (Figure S4A,B, Supporting Information). To directly evaluate the proliferation of K14^+^ cells of WT and *Scd1^−/−^* mice, we pulsed the mice at catagen stage with BrdU for 12 h. Similar to the Ki67 staining, more BrdU^+^ proliferating cells in the upper part of the HFs and the epidermis of *Scd1^−/−^* mice were observed (Figure 4B,C). These proliferating K14^+^ cells may hold more potential to differentiate into loricrin^+^ stratum corneum cells, and the keratinization in the epidermis of *Scd1^−/−^* mice was indeed enhanced during the first hair cycle (Figure S4C,D, Supporting Information).

To decipher the molecular mechanisms that led to the activation of K14^+^ keratinocytes, we conducted RNA‐seq analysis on keratinocytes of WT and *Scd1^−/−^* mice at the catagen to telogen transition stage (P19). The Kyoto Encyclopedia of Genes and Genomes pathway analysis revealed enrichment of the PI3K‐Akt signaling pathway in *Scd1^−/−^* keratinocytes (Figure 4D). We subsequently verified that the PI3K‐Akt signaling was activated in *Scd1^−/−^* keratinocytes through western blotting analysis (Figure 4E). PI3K signaling is critical for controlling cellular proliferation, differentiation, and apoptosis.^[^ ^37^, ^38^, ^39^ ^]^ To determine the causal relationship between the hyper‐PI3K signaling in epidermal cells and alopecia phenotype, the pharmacological inhibitor of PI3K, LY294002, was topically applied to the skin of *Scd1^−/−^* mice. Clearly, PI3K inhibition decreased the differentiation and proliferation of K14^+^ basal keratinocytes, improving hair regrowth in *Scd1^−/−^* mice (Figure 4F–H). Meanwhile, the skin conditions of *Scd1^−/−^* mice were dramatically improved by LY294002 treatment, accompanied by HF shortening and epidermis thinning (Figure 4I–J). We also detected integrin *α*6^high^CD34^+^ HFSCs and found that LY294002 partially restored bulge HFSCs (Figure 4K). Similarly, when we applied the inhibitor to *K14‐Cre;Scd1^fl/fl^* mice, an improvement in hair growth was observed (Figure 4L). Therefore, the cutaneous abnormalities resulting from *Scd1* deficiency are mediated by the hyperactivation of the PI3K signaling pathway in K14^+^ basal keratinocytes and their suppression on the bulge formation and HFSC localization.

### 2.5 Reduction in Hemidesmosome Assembly Is Responsible for the Hyperactivity of *Scd1^−/−^* K14^+^ Cells by Regulating Integrin *α*6‐FAK‐PI3K Signaling

**Figure 5:**
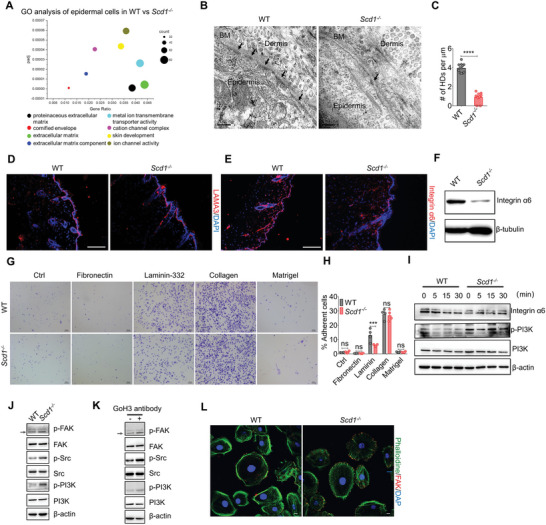
Reduction in HD assembly is responsible for the hyperactivity of K14^+^ cells with *Scd1* deficiency by regulating integrin *α*6‐FAK‐PI3K signaling. A) Gene ontology (GO) enrichment analysis of significantly dysregulated genes in epidermal cells of WT and *Scd1^−/−^* mice. B) Representative ultrastructural images of basal epidermal cells of WT and *Scd1^−/−^*, as assessed by TEM. Arrows, HDs. BM, basement membrane. Scale bars: 200 nm. C) Quantification of HDs of basal epidermal cells from WT and *Scd1^−/−^* (*n* = 12; two‐tailed unpaired *t*‐test). D) Immunostaining of LAMA3 in the skin of WT and *Scd1^−/−^* mice. Red: LAMA3; blue: DAPI. Scale bars: 130 µm. E) Immunostaining of integrin *α*6 in the skin sections of WT and *Scd1^−/−^* mice. Red: integrin *α*6; blue: DAPI. Scale bars: 130 µm. F) Western blotting analysis of integrin *α*6 in the skin of WT and *Scd1^−/−^* mice. G) In vitro cell adhesion assay. Keratinocytes from neonatal WT and *Scd1^−/−^* mice were plated on fibronectin, laminin‐332, collagen type I, or matrigel‐coated 96‐well culture plates. After 1 h, nonadherent cells were washed away and adherent cells were fixed and stained with crystal violet. Scale bars: 100 µm. H) The total covered area by adherent cells in each well was calculated using ImageJ and presented as a percentage of the total well area (*n* = 4; two‐way ANOVA with Sidak's two‐sided multiple comparisons). I) Western blotting analysis of integrin *α*6 and phosphorylated PI3K in primary keratinocytes isolated from neonatal WT, *Scd1^−/−^* or *K14‐Cre;Scd1^fl/fl^* mice and cultured for indicated time. J) Western blotting analysis of total and phosphorylated FAK, Src, and PI3K in primary keratinocytes isolated from neonatal WT mice and *Scd1^−/−^* or *K14‐Cre;Scd1^fl/fl^* mice which cultured on laminin‐332. K) Western blotting analysis of total and phosphorylated FAK and PI3K in WT primary keratinocytes cultured in plates coated with laminin‐332, with or without pretreatment of anti‐integrin *α*6 antibody, GoH3. L) Immunostaining of phalloidine and FAK in the primary keratinocytes isolated from neonatal WT and *Scd1^−/−^* mice. Scale bars: 5 µm. Data are shown as mean ± SEM. ***p < 0.001; ****p < 0.0001; ns, not significant.

To elucidate the mechanisms through which the PI3K signaling pathway was abnormally activated in *Scd1^−/−^* keratinocytes, we performed bulk RNA‐seq analysis on WT and *Scd1^−/−^* keratinocytes. Gene ontology (GO) analysis of differentially expressed genes showed that genes encoding extracellular matrix (ECM) proteins were dramatically enriched (**Figure** **5**A). Furthermore, ultrastructural studies using transmission electron microscopy (TEM) revealed that the number of electron‐dense plaques representing HDs^[^ ^40^ ^]^ was significantly reduced in the skin of *Scd1^−/−^* and *K14‐Cre;Scd1^fl/fl^* mice, compared to their littermate controls (Figure 5B,C; Figure S5A,B, Supporting Information).

HDs are essential for basal epithelial cell adhesion to the underlying basement membrane (BM). They are complexes of integrin heterodimers that interconnect with laminins in the BM and the keratin and actin cytoskeleton intracellularly.^[^ ^41^, ^42^, ^43^ ^]^ In the complex of HDs, integrin *α*6*β*4 binds to laminin‐332 (composed of laminin *α*3, *β*3, and *γ*2 chains), thereby anchoring the basal keratinocytes to the epidermal BM.^[^ ^44^, ^45^, ^46^ ^]^ To further verify the decrease of HDs in *Scd1* ^−/−^ skin, we detected the protein levels of these HD components. No marked difference in the staining of laminin *α*3 (LAMA3) was observed in the skin of WT and *Scd1^−/−^* mice (Figure 5D; Figure S5C, Supporting Information). However, a notable decrease in integrin *α*6 level was found in *Scd1^−/−^* mice (Figure 5E). Accordingly, the western blotting analysis showed lower levels of integrin *α*6 in *Scd1^−/−^* keratinocytes compared to WT controls (Figure 5F). We further performed immunofluorescence staining on serial sections of the skin of *K14‐Cre;Scd1^fl/fl^* mice and littermate controls and detected the colocalization of integrin *α*6 and *β*4. A significantly decreased expression in integrin *α*6, but not integrin *β*4 was shown in the skin of *K14‐Cre;Scd1^fl/fl^* mice (Figure S5D,E, Supporting Information). To test whether the integrin *α*6 mediated adhesion was impaired in *Scd1^−/−^* basal keratinocytes, in vitro adhesion assays were performed, with different ECM components. We found no difference in the adhesion of WT and *Scd1^−/−^* keratinocytes to fibronectin, collagen type I, or matrigel, except on laminin‐332 (Figure 5G,H). Therefore, the impairment of the interaction between integrin *α*6*β*4 and laminin‐332 contributes to the decrease of integrin *α*6 in *Scd1^−/−^* keratinocytes.

Next, we determined whether the decreased expression of integrin *α*6 in *Scd1^−/−^* keratinocytes and their impaired adhesion to laminin‐332 are responsible for the canonical PI3K signaling. When we checked the activation of PI3K in the process of adhesion on the laminin‐332 in vitro, the loss of *Scd1* in keratinocytes displayed the lower integrin *α*6 and phosphorylation of PI3K at different time points (Figure 5I). We also performed phalloidine staining on cultured *Scd1^−/−^* and WT keratinocytes to visualize the actin arrangement in cells plated on laminin‐332 in vitro. *Scd1^−/−^* keratinocytes appeared more spreading than WT cells and showed more significant variability in size, with actin stress fiber patterns rather than the cortical actin arrangement observed in WT cells (Figure S5F,G, Supporting Information). Previous studies have demonstrated that keratinocytes lacking hemidesmosomal integrin *α*6*β*4 exhibit enhanced formation in focal adhesion, cell spreading, and generation of traction force via negatively regulating the FAK‐PI3K signaling.^[^ ^47^, ^48^ ^]^ The tyrosine kinase steroid receptor coactivator (Src) can phosphorylate the Y576 and Y577 residues on FAK, leading further increment in FAK activity and recruitment of proteins that contain Src homology 2 (SH2) domains, such as that in PI3K.^[^ ^49^, ^50^, ^51^ ^]^ We, therefore, detected the phosphorylation at the Y576 and Y577 residues of FAK, Src activation, and PI3K activity in primary keratinocytes derived from WT and *Scd1^−/−^* mice. As shown in Figure 5J, *Scd1* deficiency enhanced the phosphorylation of FAK, Src, and PI3K. We further used the GoH3 antibody^[^ ^52^ ^]^ to block integrin *α*6 and mimic the impaired integrin *α*6 expression in *Scd1^−/−^* keratinocytes. Blockade of integrin *α*6 significantly decreased the adhesion of keratinocytes to laminin‐332 (Figure S5H, Supporting Information) and dramatically enhanced the phosphorylation of FAK, Src, and PI3K (Figure 5K). Additionally, combined with phalloidine, FAK staining verified the increase of focal adhesion in *Scd1^−/−^* keratinocytes plated on laminin‐332 in vitro (Figure 5L). Taken together, SCD1 exerts its effects by stabilizing the number of HDs and restricting the proliferation of keratinocytes via negative regulation of the FAK‐PI3K signaling pathway.

### 2.6 Dietary Supplementation of Oleic Acid Reverts the Cutaneous Abnormalities in Mice with *Scd1* Deficiency in Keratinocytes

**Figure 6:**
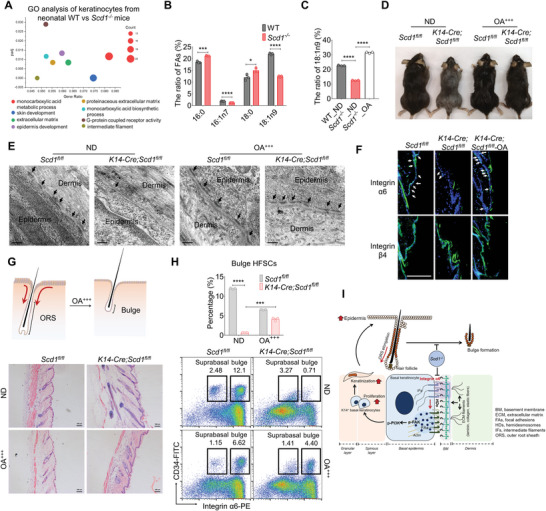
Dietary supplementation of oleic acid normalizes the keratinocyte phenotype and reverts cutaneous abnormalities in mice with *Scd1* deficiency. A) GO enrichment analysis of significantly dysregulated genes in epidermal cells of neonatal WT and *Scd1^−/−^* mice. B) The ratio of SCD1‐related fatty acids in epidermal cells of WT and *Scd1^−/−^* mice (*n* = 3; two‐tailed unpaired *t*‐test). C) The ratio of oleic acid (18:1n9) in keratinocytes from WT, *Scd1^−/−^* mice and *Scd1^−/−^* mice subjected to oleic acid (OA)‐enriched diet (OA^+++^). D) Hair coat of mice with K14‐specific deletion of *Scd1* (*K14‐Cre;Scd1^fl/fl^*) and littermate controls (*Scd1^fl/fl^*) fed with normal diet (ND) or oleic acid (OA)‐enriched diet (OA^+++^) (*n* = 5). E) Representative ultrastructural images of basal epidermal cells in *K14‐Cre;Scd1^fl/fl^* mice and littermate controls fed with ND or OA^+++^ diet as assessed by TEM. Scale bars: 0.5 µm. F) Immunostaining of integrin *α*6 and *β*4 in the skin sections of *K14‐Cre;Scd1^fl/fl^* mice and littermate controls (*Scd1^fl/fl^*) fed with normal diet or oleic acid (OA)‐enriched diet (OA^+++^) (*n* = 5). Green: integrin *α*6 (Top) and *β*4 (Bottom); blue: DAPI. Scale bars: 130 µm. G) Representative images of H&E staining of skin sections of *K14‐Cre;Scd1^fl/fl^* mice and littermate controls fed with ND or OA^+++^ diet. Scale bars: 100 µm. H) The percentage of HFSCs in *K14‐Cre;Scd1^fl/fl^* mice and littermate controls fed with ND or OA^+++^ diet as analyzed by flow cytometry. The integrin *α*6^high^CD34^+^ bulge population and integrin *α*6^low^CD34^+^ suprabasal population are shown. I) Schematic of SCD1 in the regulation of bulge formation for quiescent HFSCs during hair growth. *Scd1* deficiency impaired the expression of integrin *α*6 and the formation of HDs, thus allowing the activation of the FAK‐PI3K signaling pathway in K14^+^ keratinocytes. This alteration expedites the differentiation and proliferation of keratinocytes, resulting in downward extension of ORS and disruption of bulge formation. Data are shown as mean ± SEM. *p < 0.05; ***p < 0.001; ****p < 0.0001.

SCD1 is the rate‐limiting enzyme converting palmitic acid (16:0) and stearic acid (18:0) to yield palmitoleic acid (16:1n7) and oleic acid (OA, 18:1n9), respectively.^[^ ^53^, ^54^ ^]^ GO analysis on the bulk RNA‐seq of keratinocytes derived from *Scd1^−/−^* and WT mice enriched a series of genes related to the monocarboxylic acid metabolic process (**Figure** **6**A). Gas chromatography‐mass spectrum (GC‐MS) analysis showed that in the keratinocytes of *Scd1^−/−^* mice, there is an accumulation of SCD1 substrates (16:0 and 18:0) and a decrease in SCD1 products (16:1n7 and 18:1n9) (Figure 6B; Figure S6A,B, Supporting Information). To investigate whether *Scd1* deficiency‐mediated fatty acid desaturation disorders were the direct causes of cutaneous abnormalities, we composed a diet rich in oleic acid (diet OA^+++^), a key product of SCD1 in skin. The ratio of unsaturated fatty acids and saturated fatty acids related to SCD1 was restored in *Scd1* ^−/−^ mice fed on a diet OA^+++^ (Figure 6C; Figure S6C, Supporting Information). We found that hair growth and the number of HDs of *K14‐Cre;Scd1^fl/fl^* mice subjected to diet OA^+++^ were normalized (Figure 6D–F; Figure S6D, Supporting Information), and bulge HFSCs were significantly improved (Figure 6G,H; Figure S6E–I, Supporting Information).

In summary, *Scd1* expressed in K14^+^ cells, but not in HFSCs, maintains the formation of the bulge niche and thus favors the cyclical activity of HFSCs during hair growth. We identify a novel role of SCD1 in sustaining keratinocytes‘ expression of integrin *α*6*β*4 complex, a major component in the assemblage of HDs at the interface of epithelial cells and the basement membrane. Impaired HDs in *Scd1^−/−^* mice accelerate keratinocyte proliferation and differentiation via negative regulation between integrin *α*6*β*4 and the FAK‐PI3K signaling pathway. This effect drives the elongation of ORS, which leads to the failure to form the bulge, a quiescent niche that is essential for cyclical bouts of hair growth (Figure 6I). This study, therefore, expands on our knowledge of bulge niche biology and highlights the importance of fatty acid metabolism in maintaining the homeostasis of the bulge for HFSCs through stabilizing HDs in basal keratinocytes.

## 3 Discussion

Dynamic changes in metabolic pathways are well recognized to couple tissue‐specific stem cells with pathophysiological conditions.^[^ ^55^, ^56^, ^57^, ^58^ ^]^ Yet little is known about whether metabolic products permanently alter tissue stem cell niches and activities. Our data demonstrate that *Scd1* expression in K14^+^ cells, but not HFSCs, maintains the homeostasis of the bulge niche and, thus, supports the cyclical activity of HFSCs during hair growth. We identified a novel role of SCD1 in sustaining keratinocytes expressing integrin *α*6*β*4, a significant component in the assemblage of HDs at basal keratinocyte and BM interface. We further demonstrated that impaired HD formation in *Scd1^−/−^* keratinocytes accelerated their proliferation and differentiation via negative regulation between integrin *α*6*β*4 and the FAK‐PI3K signaling pathway.^[^ ^47^ ^]^ Subsequently, this effect drives the elongation of ORS and failure to form the bulge, a quiescent niche essential for cyclical bouts of hair growth. Dietary supplement of OA, a catabolite of SCD1, restored the homeostasis of bulge HFSCs and hair growth in *Scd1^−/−^* mice.

Since the activities of HFSCs in the dorsal skin of mice are synchronously controlled during the first few hair cycles, HF has become an ideal system for studying the interplay between stem cells and their niches. It can also help to determine the key factors driving the dynamic changes of niches required for reserving quiescent stem cells.^[^ ^10^, ^11^, ^12^, ^30^ ^]^ In our study, deficiency of *Scd1* in keratinocytes results in the failure of the first bulge formation and interrupts the maintenance of quiescent HFSCs, subsequently changing the hair growth. Indeed, the absence of bulge formation prohibits hair regeneration. A recent study has demonstrated that blocking a secondary bulge formation during hair growth induced defective HF regeneration and compromised long‐term maintenance of HFSCs.^[^ ^8^ ^]^ They found the bulge formation was related to the suppression of OXPHOS and glutaminolysis by mTORC2‐Akt signaling, a critical metabolic event in regulating the transition between ORS cells and quiescent HFSCs.^[^ ^8^ ^]^ Distinct from the defect in re‐establishing HFSC bulge in mice with mTORC2 deletion, *Scd1^−/−^* mice are lack of HFSC bulge formation in the first wave of hair growth during the postnatal period. Due to the absence of effect in *Lgr5‐Cre;Scd1^fl/fl^* mice on hair cycle, we concluded that the abnormal hair growth in *Scd1^−/−^* mice was not related to *Scd1* deficiency in HFSCs. We also excluded the lack of influence of *Scd1* deficiency in immune cells and mesenchymal cells on hair growth. Therefore, fatty acid metabolism in K14^+^ keratinocytes is vital in HFSC activity via regulating bulge formation.

Loss of *Scd1* did not affect the number of HFs. However, the deletion of *Scd1* reduced the bulge HFSCs and prevented the stem cells in the upper ORS from returning to the bulge niche to resume the HFSC state. Such restriction in *Scd1^−/−^* mice was attributed to the enhanced proliferation and differentiation of K14^+^ keratinocytes due to the reduction in the assemblage of HDs. Defects in HD components have been shown to lead to junctional epidermolysis bullosa, characterized by skin atrophy, fragility, dyspigmentation, and alopecia.^[^ ^59^ ^]^ It was recently reported that the instability of HDs, in particular through mutations in the gene encoding collagen XVII *α*1 chain (COL17A1), a component of HDs, allows basal cells to divide asymmetrically by orienting the mitotic spindle perpendicular to the underlying basement membrane, resulting in the disturbance of skin homeostasis and premature aging.^[^ ^40^ ^]^ HDs anchor basal keratinocytes to BM laminin‐332 and interconnect intracellularly with keratin intermediate filaments to form stable adhesion complexes that can withstand mechanical stress and tension. Studies have shown that the expression of HD components by basal keratinocytes fluctuates physiologically through genomic/oxidative stress‐induced proteolysis.^[^ ^40^ ^]^ In our studies, deletion of *Scd1* in keratinocytes suppressed the assemblage of HDs by impairing integrin *α*6*β*4 expression. However, how integrin *α*6*β*4 was influenced by *Scd1* deficiency remains unclear.

The clustering of integrins through the lipid raft microdomain in the plasma membrane is associated with signal transduction.^[^ ^60^, ^61^ ^]^ Through integrins, cells can sense mechanical changes in the extracellular matrix of the environment and adapt their dynamic behavior to the physical environment.^[^ ^48^, ^62^, ^63^ ^]^ The impaired assembly of HDs alters the ability of cells to withstand mechanical stress and tension, thus, affecting the proliferation, differentiation, migration, and spreading of basal keratinocytes.^[^ ^47^, ^64^ ^]^ In our studies, lowered level of integrin *α*6*β*4 expression was associated with the enhanced FAK‐PI3K activation and epidermal hyperplasia and hyperkeratosis, which likely resulted in cell crowding in the basal keratinocyte layer, and thus impede ORS shortening to form the bulge niche. We found that pharmacologically inhibiting PI3K signaling in the epidermis of *Scd1^−/−^* mice facilitates the shortening of ORS and bulge formation for reserving HFSCs, suggesting that mechanical forces may contribute to ORS shortening and bulge re‐establishment.

In conclusion, SCD1 is a critical enzyme in regulating hair growth, and the balance of fatty acid metabolism is of great significance for maintaining the homeostasis of HDs, bulge niche for quiescent HFSCs, and the kinetics of keratinocytes. This study not only reveals a novel mechanism through, which the tissue microenvironment of fatty acid metabolism regulates the homeostasis of bulge HFSCs, but also provides insights for developing new strategies for the treatment of alopecia and skin aging.

## 4 Experimental Section

### Mice

*K14‐Cre* mice were kindly provided by Dr. Xinhua Liao, Shanghai University. *Lgr5‐Cre* mice were from Dr. Jun Qin, Shanghai Institute of Nutrition and Health. *CD4‐Cre* mice [B6.Cg‐Tg(Cd4‐cre)1Cwi N9] were kindly provided by Dr. Xiaoren Zhang, Shanghai Institute of Nutrition and Health. *Lyz2‐Cre* mice [B6.129P2‐*Lyz2^tm1(cre)Ifo^*/NJU, 004781] were kindly provided by Dr. Honglin Wang, Shanghai Institute of Immunology, Shanghai Jiao Tong University School of Medicine. *Scd1^−/−^* mice (B6.129‐*Scd1^tm1Ntam^*/J, 006201), *Dermo1‐Cre* mice [B6.129×1‐*Twist2^tm1.1(cre)Dor^*/J, 008712], and tdTomato‐flox [B6Cg‐*Gt (ROSA)26Sor^tm14(CAG‐tdTomato)Hze^*/J, 007914] were purchased from the Jackson Laboratory. *Scd1^fl/fl^* mice were constructed by the Shanghai Model Organisms Center. All mice used are on the C57BL/6 background. Genetically modified sex‐matched littermates with wild‐type phenotypes were used as controls. Mice were housed in the animal facility of the Shanghai Institute of Nutrition and Health, Chinese Academy of Sciences under pathogen‐free conditions. All animal experiments were approved by the Institutional Animal Care and Use Committee of the Shanghai Institute of Nutrition and Health, Chinese Academy of Sciences.

### Cell Culture

Dorsal skin samples were collected from neonatal mice under a sterile condition and were placed dermis side down in a PBS solution containing 2 mg mL^−1^ dispase (Roche) solution at 4 °C and allowed to float overnight. Separated the epidermis and dermis layer gently, and the epidermis was allowed to float on the trypsin‐versene (Lonza) at room temperature for 20 min. Cutting the epidermis layer and the digestion was ended with a stop solution (5% FBS and 1 mm EDTA in PBS). After gentle swirling, the cells were filtered with strainers (70 mm followed by 40 mm). After washing twice with PBS, the resultant cells were seeded onto plates and cultured for 48 h. Adherent keratinocytes were expanded in a culture medium containing CnT‐PR media (CELLnTEC), 0.1% penicillin and streptomycin (Gibco), and 10 µm Y‐27632 (Sigma), and then maintained at 37 °C in a humidified incubator containing 5% CO_2_.

### Hair Cycle Induction

Dorsal hairs at the telogen phase were first shortened with a clipper followed by waxing. Hair cycle entry into the anagen phase was determined by the color of skin and reappearance of hair.^[^ ^24^ ^]^ To monitor the hair regrowth, photographs were taken with a ruler on the day of depilation (day 0) and then at the indicated time points. Hair cycle induction was quantified using intensity analysis with the ImageJ software at each time point or as a percentage of pigmented dorsal skin relative to baseline (day 0). The skin samples were harvested at different time points for histological analysis and RT‐PCR.

### PI3K Inhibitor Treatment

*Scd1^−/−^* mice were shaved dorsally at 8 to 10 weeks old. Vehicle control (DMSO in cream base) or PI3K inhibitor LY294002 (dissolved in DMSO and then added to a cream base) were topically applied on the shaved skin every other day for the duration of the experiments (3–5 weeks).^[^ ^65^ ^]^ The appearance of skin pigmentation and hair growth was monitored.

### Preparation of Paraffin Sections

For paraffin sections, dorsal skin specimens were fixed in 4% paraformaldehyde (PFA) at 4 °C overnight. After removing PFA with running tap water, the fixed tissue underwent dehydration in gradient ethanol (70% to 100%) before being transferred into xylene for clarification. The tissue samples were then immersed in melted wax and embedded in paraffin. Paraffin‐embedded skin specimens were cut into sections at 5 µm thickness for histological analysis.

### H&E Staining

Paraffin sections were deparaffinized with xylene and rehydrated from gradient ethanol (100% to 70%) to distilled water. To analyze skin structure, the nuclei and cytoplasm were stained with hematoxylin and eosin, separately. Image acquisition and further visualization were performed using ZEN Imaging software (ZEISS, Germany).

### Immunofluorescence Staining

For section staining, paraffin sections were subjected to antigen‐retrieval in a citrate acid buffer at 95 °C for 20 min. Unspecific binding of antibodies was blocked with 5% BSA. For whole‐mount staining, skin specimens were fixed for 4 h in 4% PFA at room temperature and then permeabilized for 10 min in 0.6% Triton X‐100 (PBS) and subjected to a blocking buffer (2% BSA, 0.3% Triton X‐100 in PBS) for 1 h. Tissue samples were incubated with diluted antibodies overnight at 4 °C. After washing three times with PBS, the sections were incubated with a secondary antibody for 2 h at room temperature in the dark. The nuclei were stained with DAPI. The samples were observed under confocal fluorescence microscopy (ZEISS, Germany). Image acquisition and further visualization were performed using ZEN Imaging software (ZEISS, Germany).

### Western Blotting Analysis

Cultured cells or homogenized tissues were lysed with RIPA lysis buffer (Millipore) supplemented with protease inhibitors (Roche) and 1 mm PMSF (Sigma) at 4 °C for 30 min. After clearing by centrifugation at 12 000 rpm for 15 min at 4 °C, the protein concentrations of samples were quantified by Pierce BCA protein assay kit (Thermo Fisher Scientific). After mixing with the protein loading buffer, proteins were separated by SDS/PAGE and transferred onto a PVDF nitrocellulose membrane. Unoccupied space on PVDF membrane was blocked with 5% fat‐free milk in TBST and incubated with primary antibody. HRP‐linked secondary antibody was used, and a subsequent reaction with ECL substrate (Millipore) was performed to enable detection.

### TEM

The specimen at the size of ≈1 mm^3^ was fixed in 2.5% glutaraldehyde at 4 °C overnight. After washing with PBS, the specimen was post‐fixed in 1% osmium tetroxide for 1 h, dehydrated with gradient ethanol (70% to 100%), then embedded in Epon‐812. Ultrathin sections were prepared and mounted on copper grids, stained with uranyl acetate and lead citrate, and then examined by TEM.

### Fluorescence‐Activated Cell Sorting (FACS)

Fresh dorsal skin samples were placed dermis side down on a 0.05% trypsin solution (GIBCO, 25300‐054) at 4 °C and allowed to float overnight. Epithelial cell suspensions were obtained by gently scraping the skin epidermis side with a scalpel. The cells were then filtered with strainers (70 mm followed by 40 mm). Cells were stained for 30 min with fluorescent‐dye conjugated labeled antibodies and then washed. Cell analysis and isolations were performed on a BD FACSCalibur Flow Cytometer controlled by CellQuest Pro software (BD bioscience). Flow cytometric analysis was performed using FlowJo software (FlowJo LLC).

### Cell Adhesion Assay

Ninety‐six‐well microplates were coated at 4 °C overnight with different components of ECM, including 5 µg mL^−1^ fibronectin, 30 µg ml type I collagen, 15 µg mL^−1^ laminin‐332, or 30 µg mL^−1^ Matrigel in PBS. After washing with PBS, wells were blocked by incubation with 0.5 mg mL^−1^ BSA in PBS for 10 min at 37 °C. Subsequently, wells were washed with PBS, and keratinocytes were seeded at a density of 10^5^ cells per well and incubated at 37 °C for 1 h. After removing the nonadherent cells by washing, the adherent cells were fixed with 4% PFA for 10 min, washed three times with water, and stained for 10 min with 0.2% crystal violet. Next, the plates were washed with water and air‐dried at 37 °C. The samples were observed under an optical microscope (ZEISS, Germany). Image acquisition and further visualization were performed using ZEN Imaging software (ZEISS, Germany). The total area of individual adherent cells covering each well was calculated using ImageJ software (NIH, USA).

### GC‐MS for Fatty Acids Profiles Analysis

Epithelial cells were isolated and lysed with RIPA. Internal standards were then mixed with cell lysate, and the mixture was extracted with chloroform and methyl alcohol (1:1, v/v), followed by the addition of sodium chloride. After mixing for 10 min at 2500 rpm, the lower layer of liquid was aspirated into a new tube and dried with nitrogen. The samples were dissolved in freshly prepared methylation reagent containing H_2_SO_4_/MeOH (1:25, v/v) by mixing for 10 min at 2500 rpm and incubated at 80 °C for 2 h. After cooling, samples were mixed with n‐hexane and distilled water by mixing for 10 min at 2500 rpm and then evaporated the supernatant with nitrogen. The fatty acids extraction was finally dissolved with isooctane and prepared for analysis. Fatty acid analysis was performed on an Agilent 6890N GC with a 5975B inert XL EI/CI MSD (Agilent).

### Gene Expression Analysis

For gene expression analysis, total RNA in tissue and cell samples, were extracted upon homogenization using Trizol reagent (Thermo Fisher Scientific), then subjected to a reverse transcription using the PrimeScript RT master mix (Takara, Kusatsu, Japan). Quantitative real‐time PCR was performed with FastStart Universal SYBR Green (Roche Applied Science, Indianapolis, IN) as an indicator on the 7900HT Fast Real‐time PCR system (Thermo Fisher Scientific). Detailed primers are listed in the table of the Supporting Information. For RNA‐seq, sequencing libraries were generated using NEBNext UltraTM RNA Library Prep Kit for Illumina (NEB, USA) following the manufacturer's recommendations and index codes were added to attribute sequences to each sample. Then samples were sequenced on the Illumina HiSeq platform. Sequencing reads were mapped to the mouse reference genome using Hisat2 v2.0.5. Normalization and identification of differentially expressed genes were performed using DESeq2. The resulting *p*‐values were adjusted using Benjamini and Hochberg's approach for controlling the false discovery rate. Genes with an adjusted *p*‐value < 0.05 and │log2(foldchange)│> 0 were assigned as differentially expressed.

### Statistical Analysis

Statistical analyses were performed with Prism software (GraphPad Prism 6 or 7). Data were shown as mean ± SEM. The significance was determined by a two‐tailed unpaired Student *t*‐test and Two‐way ANOVA with Sidak's two‐sided multiple. *p < 0.05; **p < 0.01; ***p < 0.001; ****p < 0.0001; ns, not significant. The exact statistical parameters were presented in respective figure legends.

## Conflict of Interest

The authors declare no conflict of interest.

## Author Contributions

Y.X. designed and performed the experiments, analyzed the data, and prepared the manuscript. L.L., K.L., Q.L., M.H., J.Y., J.C., J.Z., F.Z., Y.W., T.Z., and C.G. helped conduct the experiment. L.D., G.W., and X.W. made comments and suggestions. L.L. and Q.L. assisted in constructing manuscript. Y.W. and Y.S. led the project, guided, and edited the manuscript.

## Supporting information

Supporting Information

## Data Availability

All the data supporting the conclusions of this article are included within the article and in the Supporting Information of this the article. The sequencing data that support the findings of this study have been deposited in the Gene Expression Omnibus (GEO) with the accession code GSE184346.
