# Correction to “SCD1 Sustains Homeostasis of Bulge Niche via Maintaining Hemidesmosomes in Basal Keratinocytes”

**DOI:** 10.1002/advs.202507247

**Published:** 2025-06-10

Xue Y, Lin L, Li Q, Liu K, Hu M, Ye J, Cao J, Zhai J, Zheng F, Wang Y, Zhang T, Du L, Gao C, Wang G, Wang X, Qin J, Liao X, Kong X, Sorokin L, Shi Y, Wang Y. SCD1 Sustains Homeostasis of Bulge Niche via Maintaining Hemidesmosomes in Basal Keratinocytes. *Adv Sci (Weinh)*. 2023 Feb; 10(4):e2201949. https://doi.org/10.1002/advs.202201949.

In Figure 5 of the original publication, the panel K: p‐Src/ Src blot was incorrectly inserted. The correct image of Figure 5K should be as follows:


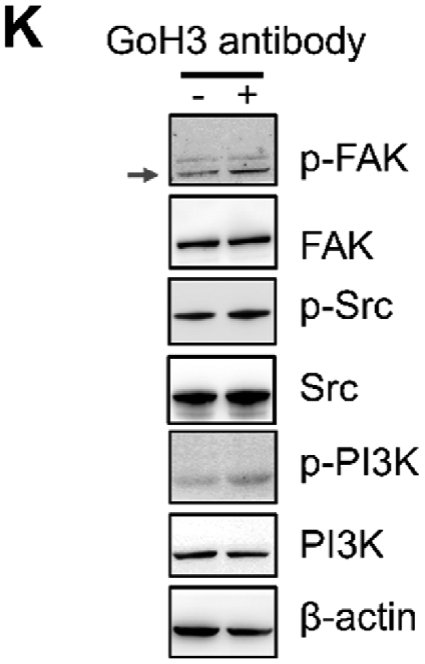


In Figure S2 (Supporting Information) of the original publication, panel K was incorrect. The correct image of Figure S2K (Supporting Information) should be as follows:


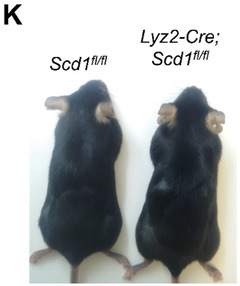


We apologize for the errors.

