# Supplementary material for: SCD1 Sustains Homeostasis of Bulge Niche via Maintaining Hemidesmosomes in Basal Keratinocytes
Source: Adv Sci (Weinh). 2022 Dec 11;10(4):2201949. doi: 10.1002/advs.202201949 (PMC9896058; doi:10.1002/advs.202201949)
Supplement: Supplementary file 1 — Supporting Information [file ADVS-10-2201949-s001.pdf]

ADVANCED SCIENCE
Open Access

Supporting Information

for *Adv. Sci.*, DOI 10.1002/advs.202201949

SCD1 Sustains Homeostasis of Bulge Niche via Maintaining Hemidesmosomes in Basal Keratinocytes

Yueqing Xue, Liangyu Lin, Qing Li, Keli Liu, Mingyuan Hu, Jiayin Ye, Jianchang Cao, Jingjie Zhai, Fanjun Zheng, Yu Wang, Tao Zhang, Liming Du, Cheng Gao, Guan Wang, Xuefeng Wang, Jun Qin, Xinhua Liao, Xiangyin Kong, Lydia Sorokin, Yufang Shi and Ying Wang**

ADVANCED SCIENCE
Open Access

Supporting Information

for *Adv. Sci.*, DOI 10.1002/advs.202201949

SCD1 Sustains Homeostasis of Bulge Niche via Maintaining Hemidesmosomes in Basal Keratinocytes

Yueqing Xue, Liangyu Lin, Qing Li, Keli Liu, Mingyuan Hu, Jiayin Ye, Jianchang Cao, Jingjie Zhai, Fanjun Zheng, Yu Wang, Tao Zhang, Liming Du, Cheng Gao, Guan Wang, Xuefeng Wang, Jun Qin, Xinhua Liao, Xiangyin Kong, Lydia Sorokin, Yufang Shi and Ying Wang**

Supporting Information

SCD1 Sustains Homeostasis of Bulge Niche via Maintaining Hemidesmosomes in Basal Keratinocytes

Yueqing Xue, Liangyu Lin, Qing Li, Keli Liu, Mingyuan Hu, Jiayin Ye, Jianchang Cao, Jingjie Zhai, Fanjun Zheng, Yu Wang, Tao Zhang, Liming Du, Cheng Gao, Guan Wang, Xuefeng Wang, Jun Qin, Xinhua Liao, Xiangyin Kong, Lydia Sorokin, Yufang Shi, and Ying Wang**

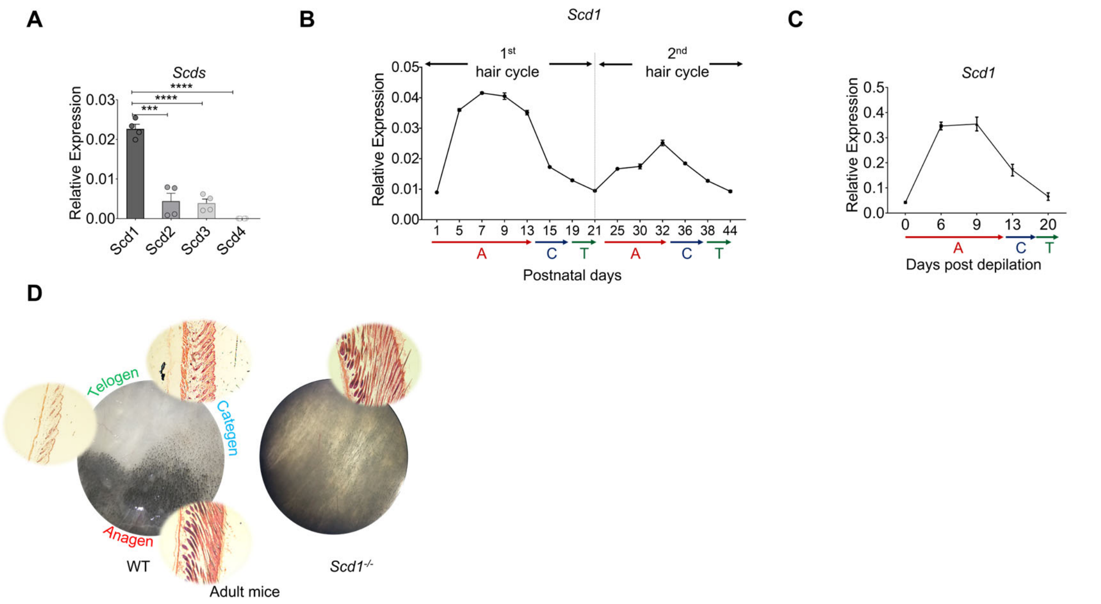

Figure S1. The expression levels of *Scd1* in the skin and the elongation of HFs in *Scd1*$^{-/-}$ mice.

(A) The mRNA levels of the four *Scds* in the skin of WT mice, as detected by RT-PCR (n=4; two-tailed unpaired *t*-test).

(B) The kinetics of *Scd1* expression at different time points during skin's first two hair growth cycles as determined by RT-PCR (n=3). A: Anagen; C: Catagen; T: Telogen.

(C) The kinetics of *Scd1* expression at different time points after hair plucking by waxing to artificially induce the hair cycle in WT adult mice (n=4). A: Anagen; C: Catagen; T: Telogen.

(D) The HFs in the skin of WT and *Scd1*$^{-/-}$ mice under a stereomicroscope.

Data are shown as mean ± SEM. ***p<0.001; ****p<0.0001; ns, not significant.

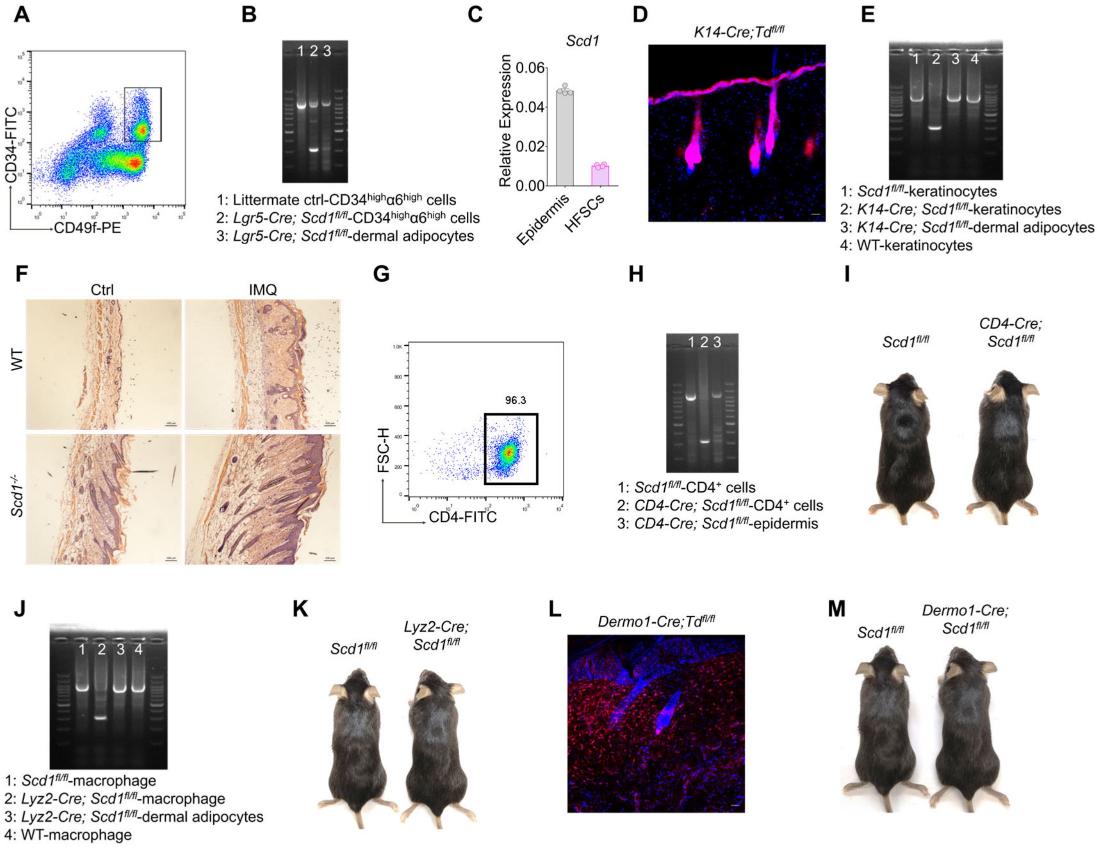

Figure S2. ***Scd1*** **deficiency in immune and mesenchymal cells do not influence hair growth.**

(A) Gating strategies of HFSCs during fluorescence-activated cell sorting.

(B) Gel electrophoresis confirmed the knockout efficiency of *Scd1* in HFSCs isolated from *Lgr5-Cre;Scd1$^{fl/fl}$* mice and littermate controls (*Scd1$^{fl/fl}$*).

(C) Expression of *Scd1* in keratinocytes and sorted HFSCs of WT mice (n=4).

(D) Representative skin whole-mount images of K14^{+} cells in *K14-Cre;Td$^{fl/fl}$* mice. Red: Td; blue: DAPI. Scale bars, 20 μm.

(E) Gel electrophoresis confirmed the knockout efficiency of Scd1 in keratinocytes isolated from *K14-Cre;Scd1$^{fl/fl}$* mice and littermate controls (*Scd1$^{fl/fl}$*).

(F) Representative images of H&E staining of skin sections from WT and *Scd1$^{-/-}$* mice with or without IMQ treatment.

(G) Purity of CD4^{+} T cells isolated from *CD4-Cre;Scd1$^{fl/fl}$* mice with microbeads by direct magnetic labeling.

(H) Gel electrophoresis confirmed the knockout efficiency of Scd1 in CD4^{+} T cells isolated from *CD4-Cre;Scd1$^{fl/fl}$* mice and littermate controls (*Scd1$^{fl/fl}$*).

(I) Hair coat of mice with CD4-specific deletion of *Scd1* (*CD4-Cre;Scd1$^{fl/fl}$*) and littermate controls (*Scd1$^{fl/fl}$*).

(J) Gel electrophoresis confirmed the knockout efficiency of *Scd1* in macrophages isolated from *Lyz2-Cre;Scd1$^{fl/fl}$* mice and littermate controls (*Scd1$^{fl/fl}$*).

(K) The hair coat of mice with myeloid-specific deletion of *Scd1* (*Lyz2-Cre;Scd1$^{fl/fl}$*) and littermate controls (*Scd1$^{fl/fl}$*).

(L) Representative skin whole-mount images of Dermo1^{+} cells in *Dermo1-Cre;Td$^{fl/fl}$* mice. Red: Td; blue: DAPI. Scale bars, 20 μm.

(M) Hair coat of mice with mesoderm-specific deletion of *Scd1* (*Dermo1-Cre;Scd1$^{fl/fl}$*) and littermate controls (*Scd1$^{fl/fl}$*).

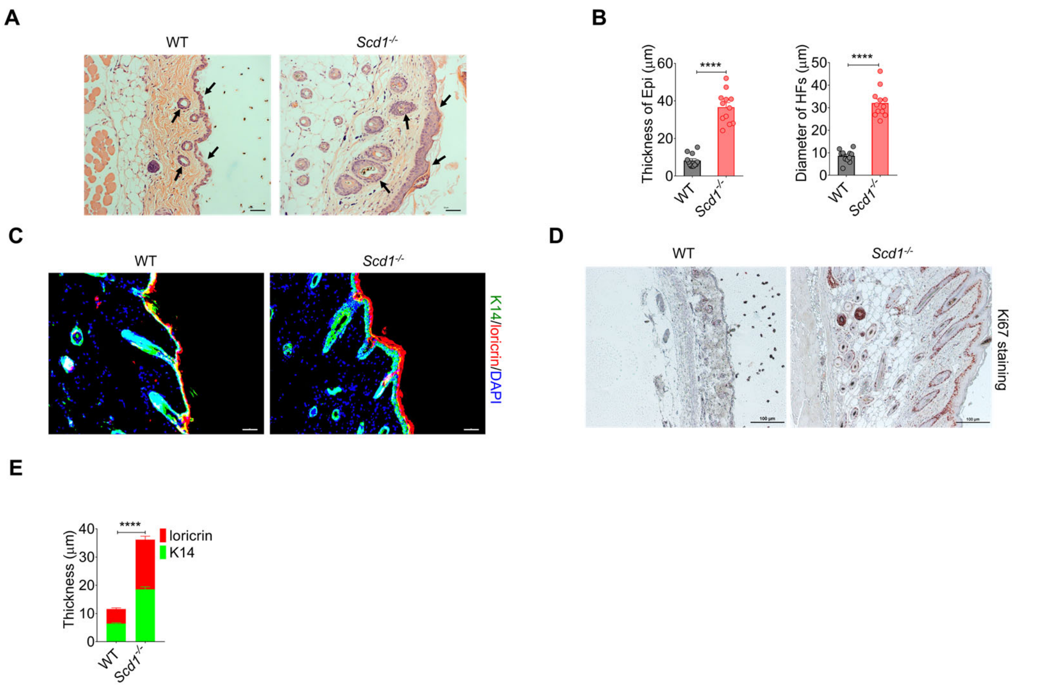

Figure S3. *Scd1* deficiency increases the proliferation and differentiation of basal keratinocytes in adult mice.

(A) Representative images of H&E staining of a transverse section of HFs in adult WT and *Scd1*$^{-/-}$ mice. Arrows indicate the excessive keratinization of HFs and epidermis. Scale bars, 50 μm.

(B) Quantification of the thickness of the epidermis (left) (n=12; two-tailed unpaired *t*-test), and the diameter of the HFs (right) (n=12; two-tailed unpaired *t*-test).

(C) Immunostaining of loricrin and K14 in keratinocytes of WT and *Scd1*$^{-/-}$ mice. Red: loricrin; green: K14; blue: DAPI. Scale bars, 50 μm.

(D) Immunostaining of Ki67 in the skin of WT and *Scd1*$^{-/-}$ mice. Scale bars, 100 μm.

(E) Quantification of the thickness of K14^{+} basal layer and loricrin^{+} differentiation layer in panel (C) (n=12; Two-way ANOVA with Sidak's two-sided multiple comparisons).

Data are shown as mean ± SEM. ****p<0.0001.

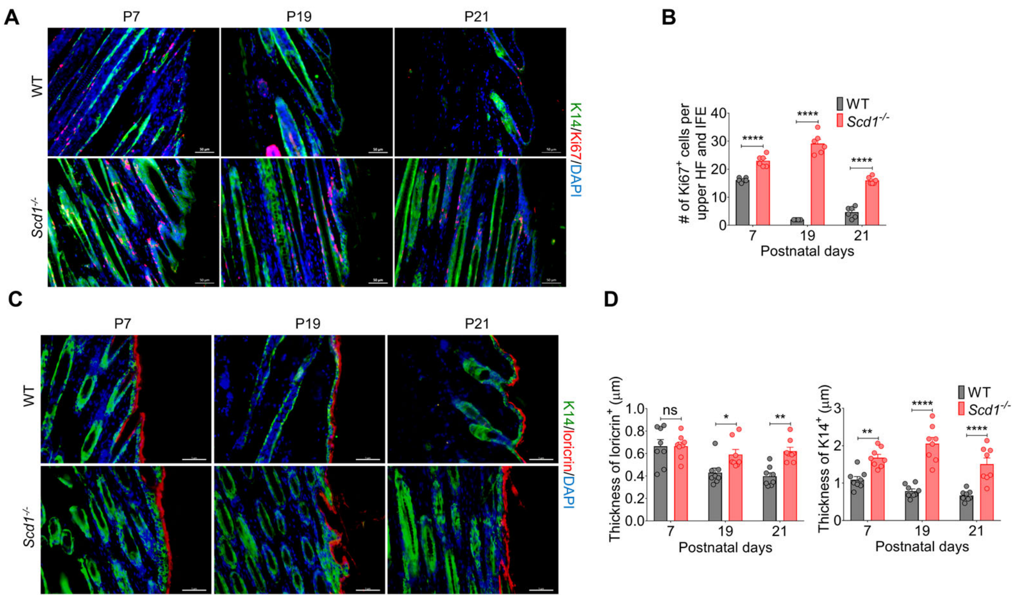

Figure S4. *Scd1* deficiency increases the proliferation and differentiation of basal keratinocytes during the first hair cycle.

(A) Immunostaining of Ki67 in the $K14^+$ epidermal cells of WT and $Scd1^{-/-}$ mice at the three phases of the first hair cycle: anagen (postnatal day 7, P7), catagen (postnatal day 19, P19), and telogen (postnatal day 21, P21). Red: Ki67; green: K14; blue: DAPI. Scale bars, 50 µm.

(B) Enumeration of $Ki67^+$ cells per upper HF in panel (A) (n=6; Two-way ANOVA with Sidak's two-sided multiple comparisons).

(C) Immunostaining of loricrin and K14 in keratinocytes of WT and $Scd1^{-/-}$ mice during the three phases of the first hair cycle: anagen (P7), catagen (P19), and telogen (P21). Red: loricrin; green: K14; blue: DAPI. Scale bars, 50 µm.

(D) Quantification of the thickness of $loricrin^+$ layer (left) (n=8; Two-way ANOVA with Sidak's two-sided multiple comparisons) and $K14^+$ keratinocytes (right) (n=8; Two-way ANOVA with Sidak's two-sided multiple comparisons) in panel (C).

Data are shown as mean ± SEM. *p<0.05; **p<0.01; ****p<0.0001; ns, not significant.

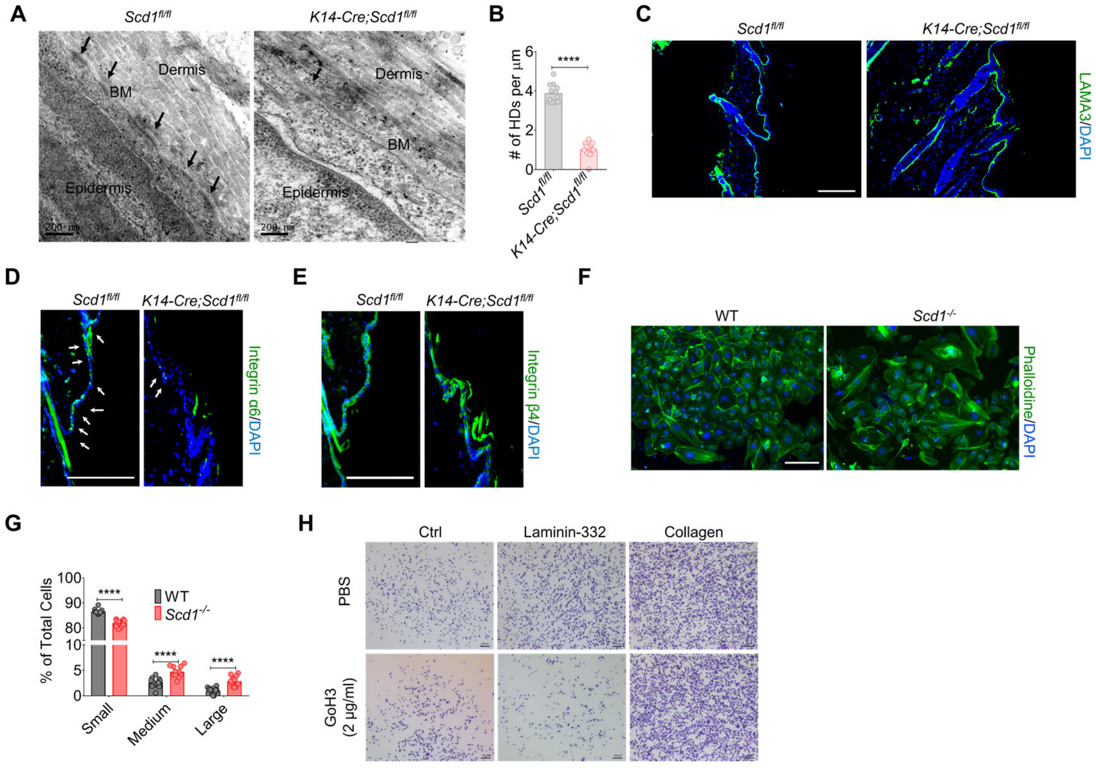

Figure S5. Loss of *Scd1* impairs integrin α6β4 mediated adhesion to laminin-332.

(A) Representative ultrastructural images of basal epidermal cells of *K14-Cre;Scd1*$^{fl/fl}$ mice and *Scd1*$^{fl/fl}$ mice, as assessed by TEM. Arrows, HDs. BM, basement membrane. Scale bars, 200 nm.

(B) Quantification of HDs of basal epidermal cells from *K14-Cre;Scd1*$^{fl/fl}$ mice and *Scd1*$^{fl/fl}$ mice (n=12; two-tailed unpaired t-test).

(C) Immunostaining of LAMA3 in the skin of *K14-Cre;Scd1*$^{fl/fl}$ mice and *Scd1*$^{fl/fl}$ mice. Green: LAMA3; blue: DAPI. Scale bars, 130 µm.

(D) Immunostaining of integrin α6 in the skin sections of *K14-Cre;Scd1*$^{fl/fl}$ mice and *Scd1*$^{fl/fl}$ mice. Green: integrin α6; blue: DAPI. Scale bars, 130 µm.

(E) Immunostaining of integrin β4 in the skin sections of *K14-Cre;Scd1*$^{fl/fl}$ mice and *Scd1*$^{fl/fl}$ mice. Green: integrin β4; blue: DAPI. Scale bars, 130 µm.

(F) Immunostaining of phalloidine in the primary keratinocytes isolated from neonatal WT and *Scd1*$^{-/-}$ mice. Scale bars, 130 µm.

(G) The percentage of cells with different sizes in primary keratinocytes isolated from neonatal WT and *Scd1*$^{-/-}$ mice (n=12; Two-way ANOVA with Sidak's two-sided

multiple comparisons).

(H) *In vitro* cell adhesion assay. Keratinocytes blocked by anti-integrin α6 antibody, GoH3, were plated on laminin-332 and collagen type I-coated 96-well culture plates. After 1 hr, nonadherent cells were washed away and adherent cells were fixed and stained with crystal violet (n=3). Scale bars, 100 μm.

Data are shown as mean ± SEM. ****$p<0.0001$.

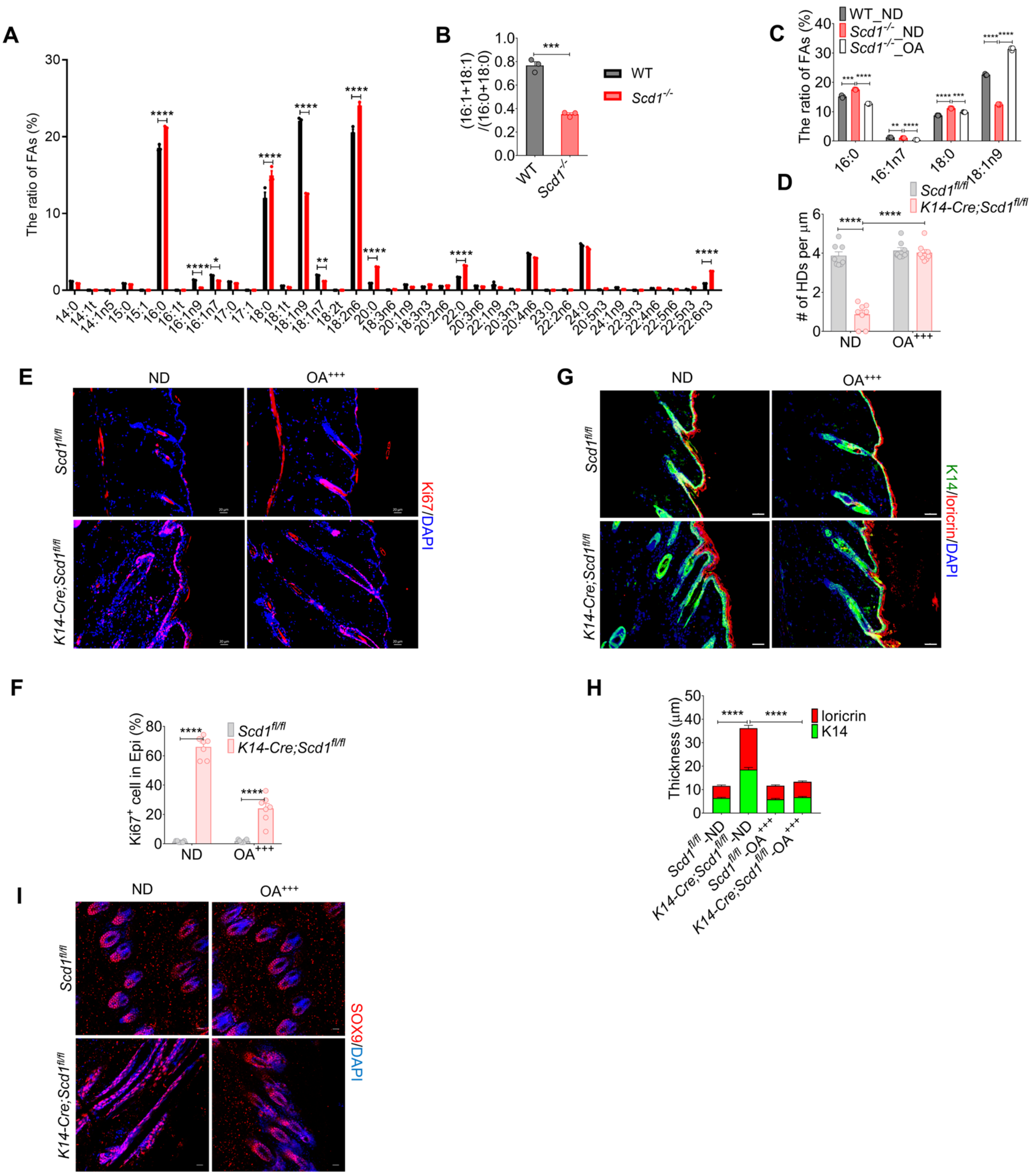

Figure S6. The changes of fatty acids in keratinocytes of *Scd1*$^{-/-}$ mice led to the cutaneous abnormalities.

(A) The ratio of fatty acids in keratinocytes from WT and *Scd1*$^{-/-}$ mice was analyzed by Gas chromatography-mass spectrometry (GC-MS) (n=3; Two-way ANOVA with Sidak's two-sided multiple comparisons).

(B) The ratio of monounsaturated fatty acids (C18:1 and C16:1) to saturated fatty acids (C18:0 and C16:0) in keratinocytes from WT and *Scd1*$^{-/-}$ mice (n=3; two-tailed unpaired *t*-test).

(C) The ratio of monounsaturated fatty acids (C18:1 and C16:1) to saturated fatty

acids (C18:0 and C16:0) in keratinocytes from WT, *Scd1*$^{-/-}$ mice and *Scd1*$^{-/-}$ mice subjected to OA^{+++} diet (n=3; two-tailed unpaired *t*-test).

(D) Quantification of HDs of basal epidermal cells from *K14-Cre;Scd1*$^{fl/fl}$ mice and *Scd1*$^{fl/fl}$ mice fed with ND or OA^{+++} diet (n=8; two-tailed unpaired t-test).

(E) Immunostaining of Ki67 in the K14^{+} epidermal cells of *K14-Cre;Scd1*$^{fl/fl}$ mice and littermate controls fed with ND or OA^{+++} diet. Red: Ki67; blue: DAPI. Scale bars, 20 μm.

(F) Quantification of the number of Ki67^{+} cells among the epidermal cells in panel (D) (n=7; two-tailed unpaired *t*-test).

(G) Immunostaining of loricrin and K14 in epidermal cells of *K14-Cre;Scd1*$^{fl/fl}$ mice and littermate controls fed with ND or OA^{+++} diet. Red: loricrin; green: K14; blue: DAPI. Scale bars, 50 μm.

(H) Quantification of the thickness of K14^{+} basal layer and the differentiating loricrin^{+} cell layer in panel (F) (n=12; two-tailed unpaired *t*-test).

(I) Representative skin whole-mount images of bulge formation in *K14-Cre;Scd1*$^{fl/fl}$ mice and littermate controls fed with ND or OA^{+++} diet, as determined by staining with a SOX9 specific antibody.

Data are shown as mean ± SEM. *p<0.05; **p<0.01; ***p<0.001; ****p<0.0001.

Supplementary Table: Primers used in the study.

Gene names	Primer Details
m-Rps18-F	GATGGGAAGTACAGCCAGGT
m-Rps18-R	TTTCTTCAGCCTCTCCAGGT
m-Scd1-F	TTCTTGCGATACACTCTGGTGC
m-Scd1-R	CGGGATTGAATGTTCTTGTCGT
m-Scd2-F	GCATTTGGGAGCCTTGTACG
m-Scd2-R	AGCCGTGCCTTGTATGTTCTG
m-Scd3-F	CAGCCCCAAACGCCACAACTT
m-Scd3-R	GATCTCGGGCCCATTCATACACG
m-Scd4-F	GCCCACTTGCCACAAGAGAT
m-Scd4-R	GTAGCTGGGGTCATACAGATCA
m-Sox9-F	GAGCCGGATCTGAAGAGGGA
m-Sox9-R	GCTTGACGTGTGGCTTGTTC
m-Lgr5-F	CCTACTCGAAGACTTACCCAGT
m-Lgr5-R	GCATTGGGGTGAATGATAGCA
m-Scd1-F for Cre loxp system	CGTCTTCAGACACACCAGAA
m-Scd1-R for Cre loxp system	CTACTCAGTGAGATCTTATGTCAG
